# A Case of Non-ketotic Hyperglycemic Hemichorea and Fahr Syndrome

**DOI:** 10.7759/cureus.60265

**Published:** 2024-05-14

**Authors:** Rebecca Oksenhendler, David Pellerin, Ahmad Almutlaq

**Affiliations:** 1 Neurology, McGill University, Montreal, CAN; 2 Neurology, King Fahad Medical City, Riyadh, SAU

**Keywords:** diabetic chorea, brain calcifications, fahr’s disease or fahr’s syndrome, diabetic striatopathy, hyperglycemic non-ketotic hemichorea

## Abstract

Non-ketotic hyperglycemic hemichorea (NHH) denotes acute hemichorea or hemiballism in patients with poorly controlled diabetes with striatal abnormalities seen on brain MRI. Here, we describe a case with diabetes mellitus and primary hypoparathyroidism who developed NHH with bilateral chorea due to the abrupt stopping of her diabetic regimen. She presented with subacute and progressive bilateral asymmetric chorea. Over the prior six months, she stopped following her diabetic regimen. Brain imaging showed features of diffuse brain calcifications suggestive of Fahr syndrome. Extensive blood investigations including genetic testing for causes of basal ganglia calcifications were unremarkable. Treatment with tetrabenazine and resumption of her diabetes medications slowly improved her chorea. This case highlights the importance of interpreting imaging findings in the context of the nature and time course of the chorea presentation. In addition, it emphasizes a systematic approach to interpreting diffuse brain calcifications with the appropriate investigations.

## Introduction

Non-ketotic hyperglycemic hemichorea (NHH) denotes acute limb hemichorea or hemiballism (rarely bilateral involvement) in patients with poorly controlled diabetes with pathognomonic striatal abnormalities on brain imaging [1]. Both chorea and ballism denote the random, involuntary, and uncontrollable jerky movements with chorea usually being distal and a smaller amplitude than ballism. NHH is not a widely recognized entity as it is usually mistaken for intracranial hemorrhage. The vast majority of NHH cases with documented ketone status were not ketotic, hence, the term “non-ketotic hyperglycemic hemichorea” [2].

Bilateral striatopallidodentate calcinosis, known as Fahr disease (an idiopathic or genetic disorder of calcium deposition abnormalities in the absence of a secondary cause) or Fahr syndrome (can be associated with calcium metabolism abnormalities due to hypoparathyroidism, infections such as human immunodeficiency virus (HIV) and congenital infections, toxin exposure, and autoimmune disorders), is associated with bilaterally symmetric calcifications [1].

## Case presentation

A 59-year-old woman with insulin-dependent type II diabetes mellitus and primary hypoparathyroidism presented with a four-week history of subacute and progressive bilateral asymmetric chorea. She was previously fully independent in her activities of daily living and instrumental activities of daily living. The chorea first began in her right hand and progressed over a month to include her arms, legs, and orofacial region. Over the last six months, she had almost completely stopped following her diabetic regimen. Examination revealed choreiform movements of her limbs (worse on the left), neck, and mouth (Video 1).

**Video 1:** Choreiform movements. Examination revealed choreiform movements of her limbs (worse on the left), neck, and mouth.

**Figure 1:**
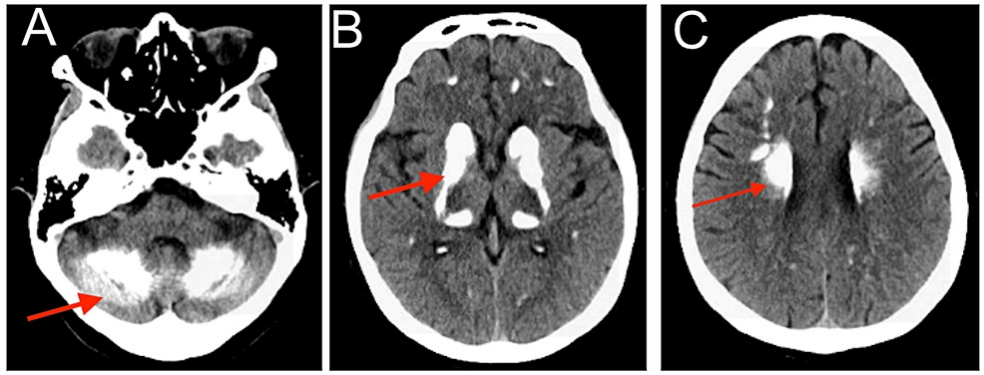
Head CT. Head CT without contrast demonstrating symmetric hyperdensities of the globus pallidi, caudate nuclei, dentate nuclei, and the periventricular and subcortical white matter (A-C, arrows). These findings along with the brain MRI were consistent with diffuse brain calcifications.

**Figure 2:**
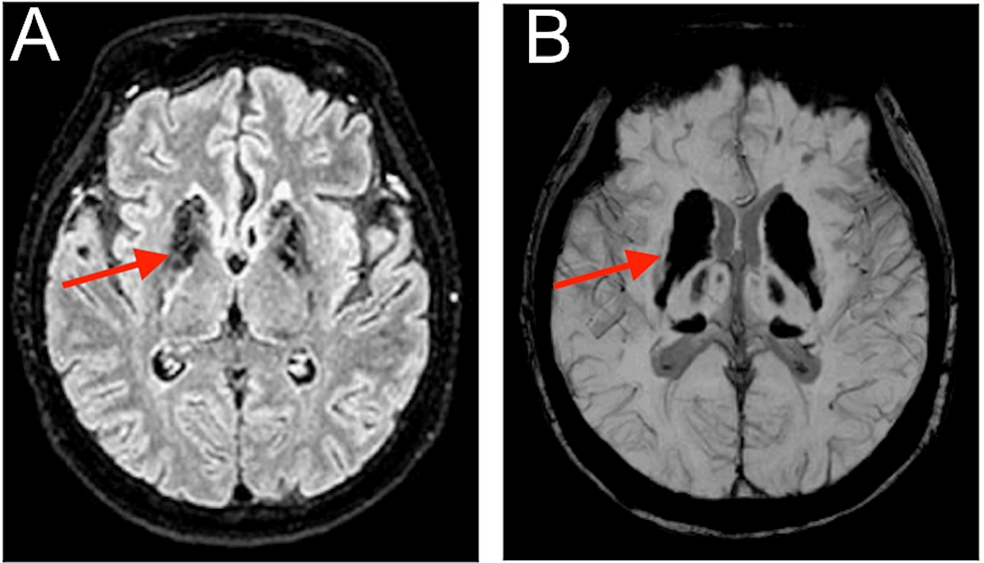
Brain MRI. Brain MRI demonstrating T2 hypointensities and blooming artifacts in these regions (A-B, arrows). These findings along with the head CT were consistent with diffuse brain calcifications.

Head CT without contrast demonstrated symmetric hyperdensities of the globus pallidi, caudate nuclei, dentate nuclei, and the periventricular and subcortical white matter (Figures 1A-1C). Brain MRI demonstrated T2 hypointensities and blooming artifacts in these regions (Figures 2A, 2B). These findings were consistent with diffuse brain calcifications.

Investigations revealed a low parathyroid hormone, a low total calcium, a high phosphate, and an elevated glycosylated hemoglobin of 12.3%. An HIV screen was negative. Peripheral blood smear did not show acanthocytes. Rheumatologic and paraneoplastic workup was negative. A commercial next-generation sequencing panel for basal ganglia calcifications was negative.

## Discussion

Bilateral striatopallidodentate calcinosis, known as Fahr disease or Fahr syndrome, is associated with bilaterally symmetric calcifications most frequently within the basal ganglia but also in the subcortical white matter, centrum semiovale, thalami, and dentate nuclei [1]. Fahr disease denotes an idiopathic or genetic disorder in the absence of a secondary cause. Fahr disease manifests in autosomal dominant, familial, and sporadic forms where *SLC20A2* is the most commonly involved gene [1]. Secondary forms can be associated with abnormalities of calcium metabolism due to hypoparathyroidism, infections such as HIV and congenital infections, toxin exposure, and autoimmune disorders [1]. Unlike age-related dystrophic calcifications which are discrete and confined to the globus pallidus, these calcifications are diffuse and form a coarse conglomerate. The presentation is variable and includes neuropsychiatric symptoms, chronic movement disorders, and cerebellar symptoms [3].

Given the subacute asymmetric progression of our patient’s chorea and recent non-compliance with her diabetes medications, her presentation was thought to be consistent with NHH. The precise mechanism for NHH is still unknown. Although most patients present with unilateral symptoms, up to 10% have bilateral limb involvement [2]. It is possible that this patient was at an increased risk of developing NHH given her basal ganglia abnormalities related to longstanding Fahr syndrome. NHH could not be confirmed on neuroimaging due to significant brain calcifications, precluding the assessment of T1 pallidal hyperintensity. She was started on tetrabenazine (a vesicular monoamine transporter 2 inhibitor) and her diabetes medications were resumed. After four months, her glycosylated hemoglobin decreased to 7.3% and her chorea slowly improved but was still noticeable. A meta-analysis study examining 49 patients with NHH found that the mean age of onset was 71 years with a female predominance [4]. The majority of cases developed bilateral movement disorder symptoms but some developed unilateral symptoms. Among the basal ganglia structures, the putamen was the most frequently involved nuclei [4]. Although some patients improve over days following proper glycemic control, many only experience resolution after several months or years [4,5].

## Conclusions

NHH denotes acute limb hemichorea or hemiballism (rarely bilateral involvement) in patients with poorly controlled diabetes with pathognomonic striatal abnormalities on brain imaging. Bilateral striatopallidodentate calcinosis, known as Fahr disease or Fahr syndrome, is associated with bilaterally symmetric calcifications.

We report the case of a patient with diabetes mellitus and primary hypoparathyroidism who developed NHH with bilateral chorea due to abruptly stopping her diabetic regimen. Overall, our case highlights the importance of interpreting imaging findings in the context of the nature and time course of the chorea presentation.
